# Podocyte Endocytosis in Regulating the Glomerular Filtration Barrier

**DOI:** 10.3389/fmed.2022.801837

**Published:** 2022-02-10

**Authors:** Xuefei Tian, Patricia Bunda, Shuta Ishibe

**Affiliations:** Department of Internal Medicine, Yale School of Medicine, New Haven, CT, United States

**Keywords:** proteinuria-nephrotic syndrome, glomerular disease, endocytosis, kidney, podocyte

## Abstract

Endocytosis is a mechanism that internalizes and recycles plasma membrane components and transmembrane receptors via vesicle formation, which is mediated by clathrin-dependent and clathrin-independent signaling pathways. Podocytes are specialized, terminally differentiated epithelial cells in the kidney, located on the outermost layer of the glomerulus. These cells play an important role in maintaining the integrity of the glomerular filtration barrier in conjunction with the adjacent basement membrane and endothelial cell layers within the glomerulus. An intact podocyte endocytic machinery appears to be necessary for maintaining podocyte function. *De novo* pathologic human genetic mutations and loss-of-function studies of critical podocyte endocytosis genes in genetically engineered mouse models suggest that this pathway contributes to the pathophysiology of development and progression of proteinuria in chronic kidney disease. Here, we review the mechanism of cellular endocytosis and its regulation in podocyte injury in the context of glomerular diseases. A thorough understanding of podocyte endocytosis may shed novel insights into its biological function in maintaining a functioning filter and offer potential targeted therapeutic strategies for proteinuric glomerular diseases.

## Introduction

The word “endocytosis” initially coined by Christian de Duve in 1963, describes the process of internalization and retrieval of extracellular material including the cellular plasma membrane components and transmembrane receptors via small vesicles. Internalized molecules from the plasma membrane can be recycled back to the cell surface or be degraded in the cell. Endocytosis orchestrates numerous cellular processes, which include membrane formation, cell signal transduction, adhesion, motility, immune response, membrane turnover, lipid homeostasis, uptake of nutrients, and development in response to the ambient environmental changes (1–3). Based on vesicular membrane components and cargoes (4) identified by electron microscopy, endocytic pathways can be broadly divided into clathrin-mediated endocytosis (5), and clathrin-independent endocytosis, which is further subdivided into caveolae-dependent endocytosis (6), phagocytosis (“cell eating”) (7, 8), macropinocytosis (“cell drinking”) (9), cholesterol-sensitive pathway (10, 11), and clathrin-independent carrier/GPI-AP-enriched early endosomal compartment (CLIC/GEEC) pathway endocytosis (12).

The kidney is composed of glomeruli, tubules, renal interstitium, and renal vasculature that work in unison to remove waste from plasma. The glomerular filtration barrier (GFB) serves as a semi-permeable membrane allowing for free filtration of small solutes while retaining larger solutes such as albumin and immunoglobulin in circulation. The GFB is composed of three layers: the podocytes located at the outermost layer facing the Bowman's space, the fenestrated endothelial cells located at the innermost layer facing the glomerular capillary lumen, and the glomerular basement membrane located between them (13). Impaired function or depletion of podocytes contributes to many forms of proteinuric chronic kidney diseases (CKD). CKD currently affects more than 800 million people worldwide and is expected to become more prevalent in the decades to come (14). Moreover, glomerular diseases account for approximately 83% of cases that progress to end-stage kidney disease (ESKD) according to the United States Renal Data system (https://adr.usrds.org/2020/).

Impaired endocytic pathways in the podocytes have been implicated in the damage of the glomerular filtration barrier. Burgeoning evidence strongly suggests that endocytic pathways play a key role in maintaining proper kidney function. Genetic missense mutations in *GAPVD1* and *ANKFY1* whose protein products interact with the endosomal regulator RAB5 (2), and missense mutations in *TBC1D8B*, whose protein product is the Rab11b-guanosine triphosphatases (GTPase)-activating protein (15) have been found causal in human steroid-resistant nephrotic syndrome. This is complimented by the genetic mouse models of disease where dysfunction in the endocytic process results in severe proteinuria suggesting its importance (Table 1). In this review, we will attempt to describe what is currently known about the podocyte endocytic process highlighting the potential mechanisms involved in maintaining a functioning filtration barrier.

**Table 1:** Loss of function of endocytosis-associated proteins in podocytes resulting in proteinuria.

**Protein**	**Characterization**	**References**
Class II phosphoinositide 3-kinase C2α (PI3KC2α)	PI3KC2α-deficient mice cause modest proteinuria, glomerulosclerosis, foot process effacement, loss of podocytes, and kidney failure	(16)
Class III phosphatidylinositol 3-kinase vacuolar protein sorting 34 (Vps34)	Podocyte-specific loss of Vps34 leads to early proteinuria, glomerulosclerosis, foot process effacement, and kidney failure	(17–19)
Dynamin 1 and Dynamin2	Loss of dynamin1 and dynamin2 in mice podocyte cause progressive proteinuria, glomerulosclerosis, foot process effacement, and kidney failure	(20)
Synaptojanin1	Loss of synpatojanin1 in mice results in severe proteinuria, foot process effacement	(20)
Endophilin1, 2 and 3	Loss of endophilin 1, 2, and 3 in mice results in severe proteinuria, foot process effacement	(20)
CD2-associated protein (CD2AP)	Loss of CD2AO exhibits proteinuria, glomerulosclerosis, foot process effacement, and kidney failure	(21)
Cyclin G-associated kinase (GAK)	Podocyte-specific loss of Gak causes progressive proteinuria, glomerulosclerosis, and kidney failure	(22)
CIN85	CIN85 exon2 deletion ameliorates proteinuria and glomerular matrix accumulation in streptozocin-induced type 1 diabetic mice; Overexpression of CIN85 in zebrafish causes severe edema, proteinuria, and effacement of foot process	(23)
Protein kinase C alpha (PKCα) and beta (PKCβ)	PKCα inhibitor stabilizes nephrin expression and ameliorates the proteinuria in streptozocin-induced type 1 diabetic mice Genetic loss of PKCα and PKCβ reduce the proteinuria in streptozocin-induced type 1 diabetic mice Dual PKCα and PKCβ inhibitor ameliorate the proteinuria in the streptozocin-induced type 1 diabetic mice and *db/db* type 2 diabetic mice	(24, 25)
Exoc5 (a central exocyst component)	Podocyte-specific loss of Exoc5 mice exhibit the massive proteinuria, mislocalization and/or loss of Neph1, Nephrin, foot process effacement and die within 4 weeks of age	(26)
Epsin	Loss of epsin1, epsin2, and epsin 3 results in progressive proteinuria, glomerulosclerosis, and kidney failure	(27)

## Principal Routes of Endocytosis

### Clathrin-Mediated Endocytosis Pathway

The hallmark of the clathrin-mediated endocytosis pathway (CME) is the involvement of clathrin, a triskelion-shaped protein that assembles in a lattice-like structure. Specific cargos in the clathrin-coated pits are internalized by dynamin-dependent vesicle fission, followed by the clathrin disassembly (28). CME allows the target proteins to be transported to endosomes, which are sorting stations directing endocytosed vesicles to either recycling or degradation pathways. The CME process progresses through a series of well-regulated steps resulting in morphological alterations. First, phosphatidylinositol-4,5-bisphosphate [PI (4,5] P2]-enriched regions in the plasma membrane recruit the clathrin adaptor proteins such as AP2, epsin, and dynamin. Second is the recruitment of proteins with Bin-Amphiphysin-Rvs (BAR)-containing domain such as endophilin, which can initiate the clathrin nucleation and polymerization resulting in the growth of the clathrin-coated pits (CCP), to approximately 60–120 nm in diameter (3, 29–31). Lastly, the formation of clathrin-coated vesicles (CCVs) is generated by the pinching off of CCP from the plasma membrane through GTPase dynamin assembly, GTP hydrolysis, dephosphorylation of [PI (4,5] P2] by synaptojanin. Lastly, uncoating of clathrin and shedding of the CCPs occurs with the help of auxillin/cyclin G-associated kinase (GAK) and hsc70 (32–34).

### Clathrin-Independent Endocytosis Pathway

In order to adapt to the requirements of different cell types, the endocytic machinery in mammals is highly complex. CME alone is likely inadequate to meet this demand (35). Many mechanisms of clathrin-independent endocytosis (CIE) that play a critical role in cell trafficking have been identified and characterized, especially with the development of electron microscopy (4). Eleven types of CIE modes were discovered and identified, including macropinocytosis, phagocytosis, caveolae-dependent endocytosis, arf6-dependent endocytosis, flotillin-dependent endocytosis, CLIC/GEEC-mediated pathway endocytosis, fast endophilin-mediated endocytosis (FEME), ultrafast endocytosis (UFE), activity-dependent bulk endocytosis (ADBE), IL-2Rβ pathway endocytosis, and massive endocytosis (4, 35, 36). Interestingly, CME and these 11 types of CIE are endocytic pathways that are cholesterol-dependent. Moreover, CME and 6 types of CIE including phagocytosis, caveolae-dependent endocytosis, FEME, ADBE, UFE, IL-2Rβ pathway endocytosis, are dynamin-dependent (35).

Caveolae-dependent endocytosis is one of the most well-described CIE pathways in cells including podocytes. Caveolae are named for their resemblance to caves or flask-shaped cholesterol-enriched smooth membrane invaginations that are 50–80 nm in diameter (37). The integral membrane protein, caveolin-1, and other adaptor proteins of the cavin family are required for this process, in which phosphorylation of caveolin-1 initiates the initial budding formation and internalization before dynamin and actin machinery-dependent fission (4, 38). Caveolae-dependent endocytic processes have been identified in the endothelial cells, epithelial cells, fibroblasts, adipocytes, and human podocytes (35, 39, 40), and are found to participate in the trafficking of sphingolipids or proteins through lipid rafts. Downregulation of Wilms tumor 1(WT1), a podocyte-specific transcription factor, mediates activation of Wnt/β-catenin signaling by recruiting LRP6 (LDL Receptor Related Protein 6) to the caveolin-dependent endocytic pathway, resulting in the downregulation of nephrin and induction of apoptosis (41). Flotillin-dependent endocytosis is associated with lipid rafts to select lipid cargo mediated by flotillin through mechanisms that are not well understood (42, 43). In addition, the role of dynamin in flotillin-dependent endocytosis also remains unclear. However, studies suggest that this pathway may serve as adaptors in other forms of CIE rather than as an individual endocytic process (35, 44). In the CLIC-GEEC endocytosis pathway, proteins such as GPI-anchored aminopeptidases (GPI-APs) are internalized in tubulovesicular structures called GPI-AP enriched early endosomal compartments (GEEC) (12, 45). The trafficking of many internalized membrane and fluid-phase contents is endocytosed through CLIC-GEEC endocytic pathway, which is a dynamin-independent (12, 35, 46). Although CLIC -GEEC pathway does not require dynamin for endocytosis, dynamin is associated with the post-internalization of GEEC (12). Many cytokine receptors are internalized by the IL-2Rβ endocytic pathway, which requires activation of the small GTPases RhoA and Rac1, as well as actin polymerization, which finally forms vesicles 50-100 nm in diameter (47, 48). The small GTPase ADP-ribosylation factor 6 (Arf6) is localized at the interface of the plasma membrane and endosomal structure (49), Arf6-GTP activates the phosphatidylinositol-4 phosphate 5-kinase, which is involved in cytoskeleton rearrangement and initiating cargo internalization including the IL-2 receptor α-subunit, and β1-integrin (50–52).

Phagocytosis engulfs particles larger than 500 nm into membrane-containing vesicles 0.5–3 μm in diameter called phagosomes. Phagocytosis is best characterized by its role in defending against microbial pathogens (35, 53). Pinocytosis uptake the contents dissolved in the fluid phase. Pinocytosis has two forms known as micropinocytosis and macropinocytosis which is based on the sizes of generated endocytosed vesicles. Pinocytosis is essential for the cellular absorption of water, nutrients, and ions in the fluid phase (35, 54). FEME is a non-constitutive mode of CIE that is triggered following stimulation of G-protein-coupled receptors and cytokine receptors through ligand binding. This process is mediated by pre-existing membrane clusters of endophilin resulting in rapid internalization (5–10 s). FEME is regulated by phosphoinositides and kinases such as Src, LRRK2, Cdk5, and GSK3β, and is dynamin-dependent (35, 55). The fundamental importance of these pathways remains elusive as genetic knockout models deleting important CIE pathways have not been shown to result in severe proteinuria.

## Endocytosis in Podocyte Homeostasis and Disease

### Role of Phosphatidylinositol 4,5-Bisphosphate in Podocyte Endocytosis

Phosphoinositides are membrane lipids generated by phosphorylation on the inositol domain. Phosphatidylinositol 4,5-bisphosphate (PI(4, 5)P2) is the most abundant phosphoinositide species enriched at inner leaflets of the cell membrane which is mainly produced by type I and type II phosphatidylinositol-phosphate kinases (PIPKs) (56). PI(4, 5)P2 can also be generated by dephosphorylation of PI(3, 4, 5)P3 mediated by the phosphatase and tensin homolog (PTEN) (3, 57). PI(4, 5)P2 plays an essential role in the regulation of CME (58). PI(4, 5)P2 promotes the initial step in the CME process by its interaction with clathrin, clathrin adaptors, and its effectors (3). Besides the critical role of PIPKs in the production of P(4, 5) P2, it also regulates poly-phosphoinositide phosphatase, synaptojanin 1, which is responsible for dephosphorylation and hydrolysis of PI(4, 5)P2 (49, 59). Global loss of synaptojanin1 enhances PI(4, 5)P2 levels and results in an accumulation of clathrin-coated vesicles (60, 61). These global knockout mice die early likely due to the inability of the pups to feed properly. Besides its neurologic importance, we have demonstrated that loss of synaptojanin1 results in proteinuria and extensive podocyte foot process effacement (20). Moreover, the importance of phosphoinositides in podocyte biology has been shown through the genetic loss of class II phosphoinositide 3-kinase C2α (PI3KC2α), an enzyme that generates PI(3)P and PI(3, 4)P2. These mutant mice develop proteinuria and kidney failure, along with altered expression of nephrin and synaptopodin in podocytes (16). Deletion of a key endosomal trafficking regulator, the class III phosphatidylinositol (PtdIns) 3-kinase vacuolar protein sorting 34 (Vps34), specifically in podocytes results in aberrant endosomal membrane morphology, early proteinuria, glomerulosclerosis, and foot process effacement (17–19). Vacuolation phenotype is observed when the Vps34 downstream effector PIKfyve is absent. This phenotype is rescued in Vps34-deficient podocytes in the presence of both Vps34 and PIKfyve (19).

Besides CME, studies have indicated that PI(4, 5)P2 may also play important roles in the regulation of CIE. Dynamin and cortactin, a PI(4, 5)P2 -binding protein, have been shown to play important roles in caveolin-dependent endocytosis through actin-modulation and plasma membrane-remodeling (62, 63). PI(4, 5) P2 is a downstream effector of GTPase ARF6, which plays an important role in the ARF6-dependent endocytosis pathway (64). Additionally, PI(4, 5)P2 plays an essential role in macropinocytosis and phagocytosis that are regulated by GTPase Rac1 (64). Yet the role of PI(4, 5)P2 in CIE has not been well documented in podocytes and serves as an area open for further studies.

### Role of Actin Cytoskeleton in Podocyte Endocytosis

The actin cytoskeleton in podocytes provides architectural and functional support that is critical for the maintenance of a normal GFB (13). The disruption of the actin cytoskeleton is often associated with podocyte damage, manifesting as massive proteinuria, which is a hallmark of many glomerular diseases and is associated with increased progression to CKD (65). Genetic mutations in actin cytoskeleton-interacting proteins have been shown to cause proteinuric CKD. Mutations such as *MYO9A* (a Rho-GTPase activating protein myosin) (66), *MYO1E* (nonmuscle class I myosin, myosin 1E) (67), *INF2* (a member of the formin family of actin-regulating proteins INF2) (68), *DAMM2* (a member of the formin family of actin-regulating proteins DAAM2) (69), *ACTN4* (alpha-actinin 4) (70), *CD2AP* (CD2-associated protein) (71), and *ANLN* (filamentous actin [F-actin] binding protein) (72) have been identified in patients with nephrotic syndrome and histological evidence of focal segmental glomerulosclerosis (FSGS). Recent evidence suggests that the actin cytoskeleton is critical for CME in cells including the podocytes and have been implicated also in CIE (35, 49).

Membrane invagination during CME is mediated by actin polymerized around the endocytic coat in mammalian cells that are dependent on the N-WASP-activated Arp 2/3 complex, an important cortical actin nucleator (73, 74). Ultrastructural examination by electron microscopy has demonstrated that the actin filaments localize to the endocytic pit at the early stages of CME while localizing between the endocytic pit and adjacent plasma membrane at the later stages. The actin cytoskeleton network around the invaginated vesicles appears to play a role in force generation by constricting and elongating the endocytic pit, thereby driving it to detach from the plasma membrane (75). The podocyte actin cytoskeleton is highly dynamic and regulated by small GTPases, but the large GTPase dynamin also plays a critical role through binding to and regulating filamentous actin, which is an essential component of the maintenance of normal podocyte foot process architecture.

**Figure 1:**
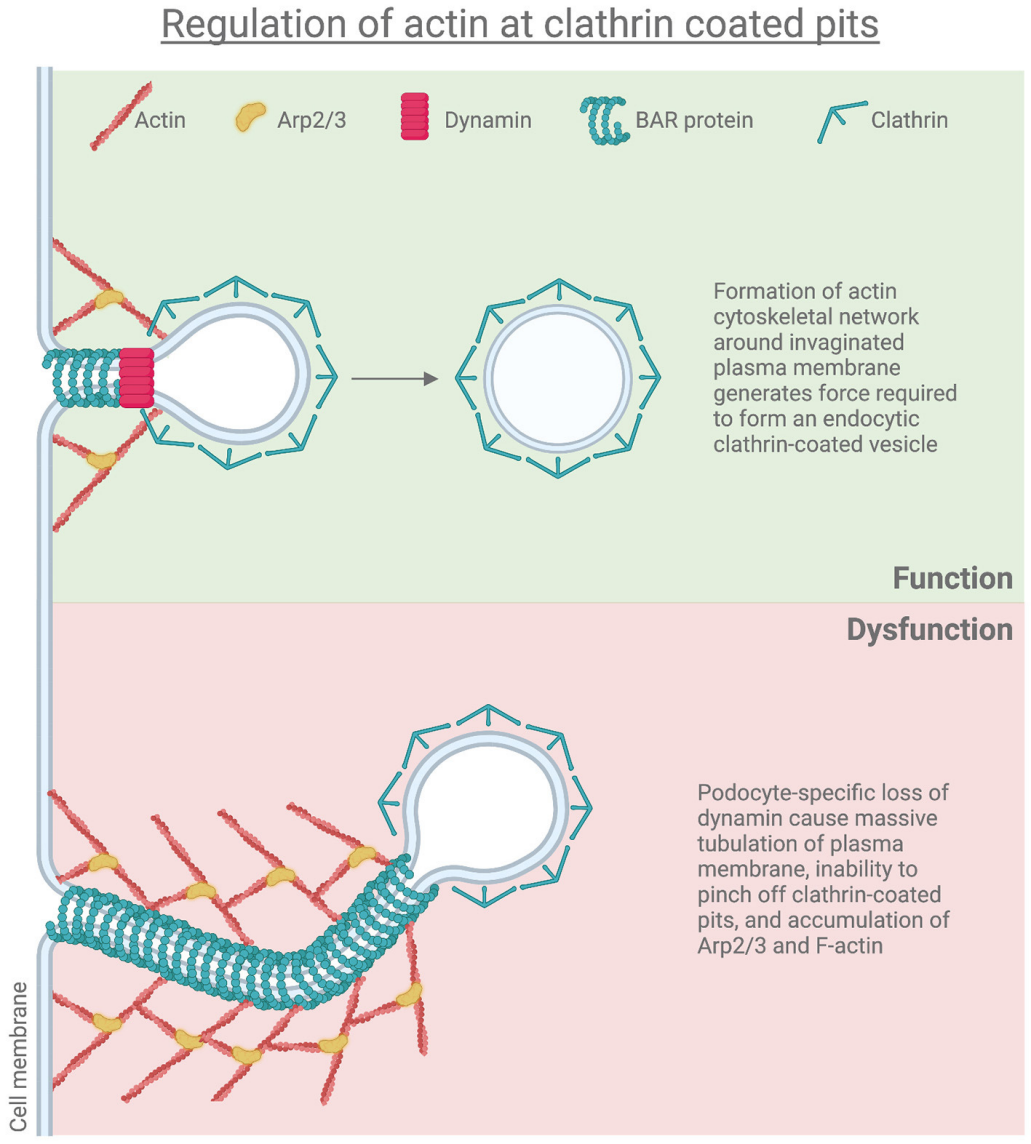
Regulation of actin at clathrin coated pits.

The importance of dynamin has been demonstrated where cathepsin-mediated dynamin proteolysis results in podocyte defects due to abnormal actin structure (76). Our studies showed that loss of both *Dnm1* and *Dnm2* specifically in mouse podocytes (*Dnm*-DKO) exhibited massive proteinuria, progressive glomerulosclerosis, and kidney failure (20). Dynamin-deficient podocytes showed an accumulation of arrested endocytic pits, which contain Arp2/3, indicating a connection between the endocytosis machinery and the actin cytoskeleton (20). Podocyte-specific loss of *Dnm1* and *Dnm2* resulted in massive tubulation of plasma membrane endocytic clathrin-coated pits with significant accumulation of Arp2/3 and F-actin in *Dnm*-DKO fibroblasts (77) (Figure 1). Furthermore, Khalil et al demonstrated that there was increased glomerular *Dnm1* and *Dnm2* expression in Dahl rats prior to the onset of proteinuria. These findings were recapitulated in increased dynamin expression in patient kidney biopsies from various proteinuric CKD including minimal change disease, FSGS, IgA nephropathy, lupus nephritis, and diabetic kidney disease. Hence, there appears to be a minimum level of dynamin that is required for the maintenance of the GFB, and increased expression of dynamins may serve as a compensatory mechanism in response to pathological conditions (78, 79).

Dynamin has also been shown to interact with the Arp2/3 (80, 81) and directly with actin (82) promoting dynamin to assemble in higher-order structures. This has led to exciting possibilities of promoting actin-dependent dynamin oligomerization by use of the small molecule Bis-T-23 to improve proteinuria and the progression of CKD in various rodent injury and genetic models (83). Mechanistically, Bis T-23 appears to promote actin stress fiber formation and focal adhesion maturation (84).

Dynamin1 has also been shown to colocalize with microtubule bundles in mouse podocytes, and loss of dynamin1 impairs microtubule formation and stabilization. Furthermore, dynamin1 and dynamin2 double knockout primary mouse podocytes showed rare microtubule-rich protrusions (85).

Non-muscle myosins have been suggested to play a key role in CME. Myosin II is required for clathrin-coated pit curvature progression and normal symmetry (86). Myosin 1E (Myo1E) is an actin-based motor protein, that interacts with dynamin and synaptojanin1 (87) and uses energy from the hydrolysis of adenosine triphosphate (ATP). Myo1E is recruited at late stages of clathrin-coated pits coincident with actin assembly and is required for N-WASP recruitment (75, 88, 89). Depletion of Myo1E inhibits transferrin endocytosis and extends the lifetimes of clathrin and dynamin on the invaginated vesicles (86, 88). Overexpression of Myo1E in the mouse podocytes promotes the expression of F-actin and increases albumin endocytosis mediated by dynamin (90). Mutations in *MYO1E* (A159P and Y695X) were reported causing autosomal recessive steroid-resistant FSGS further illustrating the importance of this gene (67).

The non-catalytic region of the tyrosine kinase (Nck) family of adaptors including Nck1 and Nck2, plays key roles in the remodeling of the cytoskeleton (91). Phosphorylation on the tyrosine residues of nephrin promotes the nephrin-Nck (Nck1/Nck2) complex formation which binds via the SH3 domain of Nck and results in actin assembly (92). Nck also interacts with GTPase dynamin, lipid phosphatase synaptojanin1, and actin-polymerizing protein N-WASP to regulate the actin assembly and endocytosis (91, 93, 94).

CD2-associated protein (CD2AP) is a cortactin-binding scaffold protein that controls actin dynamics and participates in membrane trafficking during endocytosis by interacting with dynamin, synaptojanin1, endophilin, and nephrin (49, 95). CD2AP has three Src homology 3 (SH3) domains and a coiled-coil domain contributing to its functions on actin dynamics and endocytosis by interactions with other proteins containing proline-rich domains (96). Interestingly, mice lacking *Cd2ap* develop nephrotic syndrome and die at 6–7 weeks of age due to kidney failure (21). Furthermore, it has been observed that human mutations in *CD2AP* are associated with FSGS (95, 97).

More recently, cyclin G-associated kinase (GAK), a ubiquitously expressed kinase that is associated with the uncoating of the clathrin-coated vesicles has been shown to be indispensable in podocytes (98, 99). Podocyte-specific *Gak* mice developed progressive proteinuria, glomerulosclerosis, and kidney failure, due to cytoplasmic calcium dysregulation, aberrant calpain-1/-2 activation, and actin rearrangement (22).

Although the role of the actin cytoskeleton in podocytes has not been fully understood, the actin filament accumulating around the endocytic pits provides the constriction force required for pulling the invaginated vesicles inwards (92, 100). Its importance in counteracting the membrane tension caused by the significant stretch and shear strain due to the cell adhesion to the glomerular basement membrane has been solidified. Thus, the intimate link between actin and endocytosis in podocytes plays a critical role in maintaining a normally functioning filtration barrier.

### Regulation of Slit Diaphragm in Podocyte Endocytosis

The highly specialized intracellular junctions called slit diaphragm are formed by the interdigitating foot processes of podocytes. The slit diaphragm is an essential part of the glomerular size-selective filtration barrier (13). Podocyte differentiation requires recruitment of the slit diaphragm–specific, neuronal junction proteins to membrane domains, in which endocytic trafficking plays an important role. Hence, the endocytic process regulating a subset of slit diaphragm proteins appears critical to maintaining the integrity of the GFB (87, 101).

Podocin, encoded by the *NPHS2*, is a membrane-attached protein that is required in maintaining the integrity of the podocyte slit diaphragm (102). In both the puromycin aminonucleoside nephrosis (PAN) and poor-prognosis human IgA nephropathy, endocytic dependent translocation of podocin to the cytoplasm has been demonstrated using immunofluorescence. Furthermore, podocin co-localizing with Rab5 (a key factor in regulating early endocytosis) during podocin internalization has been observed. This suggests podocin trafficking may play a critical role following podocyte injury and contributes to the disruption of the integrity of the GFB (103). The protein sorting nexin 9 (SNX9) interacts with podocin via its C-terminal BAR domain, and SNX9 expression has been shown to colocalize with podocin following adriamycin-induced podocyte injury *in vitro* and *in vivo*. Its expression is also upregulated in kidney samples of patients with severe glomerular injury. In addition, SNX9 binds directly to dynamin and stimulates dynamin assembly and GTPase activity. Hence, SNX9 may also play a prominent role in promoting podocin endocytosis by regulating dynamin (104).

Nephrin, encoded by *NPHS1*, is a podocyte-specific protein and a core component of the slit diaphragm. Proper expression of nephrin on the cell surface is critical in ensuring the integrity of the GFB. Downregulation or mislocalization of nephrin has been indicated in the pathogenesis of proteinuric kidney disease. Human mutations have been found to result in Autosomal Recessive Congenital Nephrotic Syndrome of the Finnish type (105, 106). It has been demonstrated that the long isoform of CIN85 (RukL) interacts with nephrin and mediates its endocytosis in podocytes; the paralog CD2AP can also stabilize the slit diaphragm by binding to nephrin (107). CIN85/RukL can regulate ubiquitination and several types of degradative endosomal sorting. Loss of CIN85/RukL preserves the nephrin surface expression on the podocyte slit diaphragm and reduces podocyte loss and proteinuria in the streptozotocin-induced type 1 diabetic mice. On the contrary, overexpression of CIN85/RukL in zebrafish results in severe edema and proteinuria (23). Protein kinase C (PKC) and casein kinase 2 substrate in neurons 2 (PACSIN2), also known as syndapin II, is a member of the Bin-Amphiphysin-Rvs (BAR) family that contains an evolutionary membrane binding and sculpting domain. PACSIN2 can accelerate endocytosis and intracellular trafficking of nephrin via the involvement of rabenosyn-5, which is involved in early endosome maturation and endosomal sorting (108). PKCα, one of the PKC isoforms, **has** been implicated in the endocytic process. Elevated PKCα levels in podocytes result in enhanced endocytosis of nephrin and instability of the slit diaphragm, which may play an important role in the development of albuminuria in the diabetic mice model (109). Treatment with PKCα inhibitors preserved podocyte nephrin retention and reduced proteinuria in type 1 diabetic mice (24). Thus, PKCα and PKCβ inhibitors may play a salutary role in alleviating the preexisting albuminuria in both types I and type II diabetic mice (25). The phosphotyrosine adaptor protein ShcA promotes nephrin endocytosis by increasing the tyrosine phosphorylation of nephrin resulting in aberrant nephrin turnover and distribution within the slit diaphragm (110). Podocyte ShcA expression was found to increase when co-stained with nephrin in IgA nephropathy, minimal change disease, and FSGS kidney biopsy specimens (108).

Human missense mutations of *GAPVD1* were identified in patients with early-onset steroid-resistant nephrotic syndrome using the whole-exome sequence. Mutated *GAPVD1* reduced nephrin binding and clathrin uncoating via RAB5 regulation, resulting in impaired endocytosis and nephrin mistrafficking which was confirmed by the silencing *Gapvd1* in drosophila (2). The deletions of exocyst 4 (EXOC4), one of the highly conserved octameric proteins of the exocyst trafficking complex which mediates the targeting and docking of endocytosed vesicles, was also identified in patients with nephrotic syndrome. Podocyte-specific loss of *Exoc5* (a central exocyst component interacting with Exoc4) in mice showed massive proteinuria, severe glomerulosclerosis, and fibrosis. In addition, mislocalization and/or loss of nephrin and neph1 expression were observed and this was implicated as a major contributor to this phenotype. This suggests the presence of an important mechanism that regulates the trafficking of nephrin and neph1 can help maintain an intact filtration barrier (26). Moreover, IQ domain GTPase-activating protein 1 (IQGAP1), a scaffold protein containing multiple protein-binding domains that act as a polymerization platform for a variety of proteins and signaling kinases, mediates nephrin endocytosis and has been shown to play a functional role in diabetic kidney disease (111).

Using site-directed mutagenesis, various biochemical methods, single-plane illumination microscopy, and a human nephrin-expressing zebrafish model, canonical Y*XX*Ø-type motif, Y^1139^RSL, was demonstrated as an endocytic motif for clathrin-mediated nephrin endocytosis. Y^1139^RSL motif–mediated endocytosis facilitates nephrin delivery leading to foot process organization and a functional slit diaphragm in a human nephrin-expressing zebrafish model (112). Phosphorylation (activation) and dephosphorylation (deactivation) of nephrin have been identified as an essential posttranslational event that maintains proper podocyte structure and function. Nephrin tyrosine residue phosphorylation by Src family kinases Fyn and Yes, promotes Nck adaptor protein binding mediating actin polymerization and facilitating the endocytic process (92, 113, 114). Nck's SH3 domains also play a role in coupling dynamin to nephrin, as it allows dynamin to bind through its proline-rich domain. Increased nephrin tyrosine phosphorylation upon podocyte injury correlates with a decreased membrane surface nephrin. Interestingly, mice lacking-Nck binding sites on nephrin are resistant to podocyte injury, which suggests that nephrin-Nck interaction regulates the nephrin endocytosis at the slit diaphragm (92). Nephrin has also been shown to undergo caveolin-dependent endocytosis (20) and CIE (raft-mediated endocytosis) (115). It is postulated that dephosphorylated nephrin appears to be involved in caveolin-dependent endocytosis, while phosphorylated nephrin seems to participate in the raft-mediated endocytosis (115).

Podocytes exhibit apicobasal polarity, which maintains the fundamental function of podocyte biology. The apical polarity determinants of the partitioning defective-3 (PAR-3) and atypical protein kinase C (aPKC) are in the form PAR/aPKC complex (Par3/Par6/aPKC), and this complex regulates tight junction formation and slit diaphragm assembly. The RNAi-mediated knockdown of PAR-complex proteins in Drosophila nephrocytes, a model of vertebrate podocytes, causes a severe endocytic defect due to impaired localization of aPKC at the plasma membrane. This results in impaired slit diaphragm localization. In contrast, the knockdown of Scribble, one of the lateral polarity regulators of nephrocytes, also leads to a severe reduction in endocytosis. These results suggest that apical polarity determinants are important for endocytosis and slit diaphragm homeostasis. Basolateral polarity regulators are also critical for mammalian nephrin homolog Sns localization in Drosophila (116). Basolateral polarity proteins- Discs large, Scribble, Lethal giant larvae, and Par-1 are important for normal endocytic trafficking of slit diaphragm proteins including zonula occludens-1 (ZO-1), which was confirmed by colocalization with early endosome Rab5, recycling endosome Rab7, and late endosome Rab11, respectively. The critical importance of ZO-1 in podocyte biology was recently shown when mice with podocyte-specific *Zo-1* knockout mice exhibited extensive proteinuria and kidney failure along with disruption of the slit diaphragm (117). ZO-1 is mislocalized from the plasma membrane to the cytosol following lipopolysaccharide or protamine sulfate-induced podocyte injury (118). Loss of basolateral polarity proteins led to slit diaphragm protein mislocalization into the cytoplasmic vesicles of nephrocytes. This suggests that the basolateral polarity is critical for slit diaphragm recycling (119).

**Figure 2:**
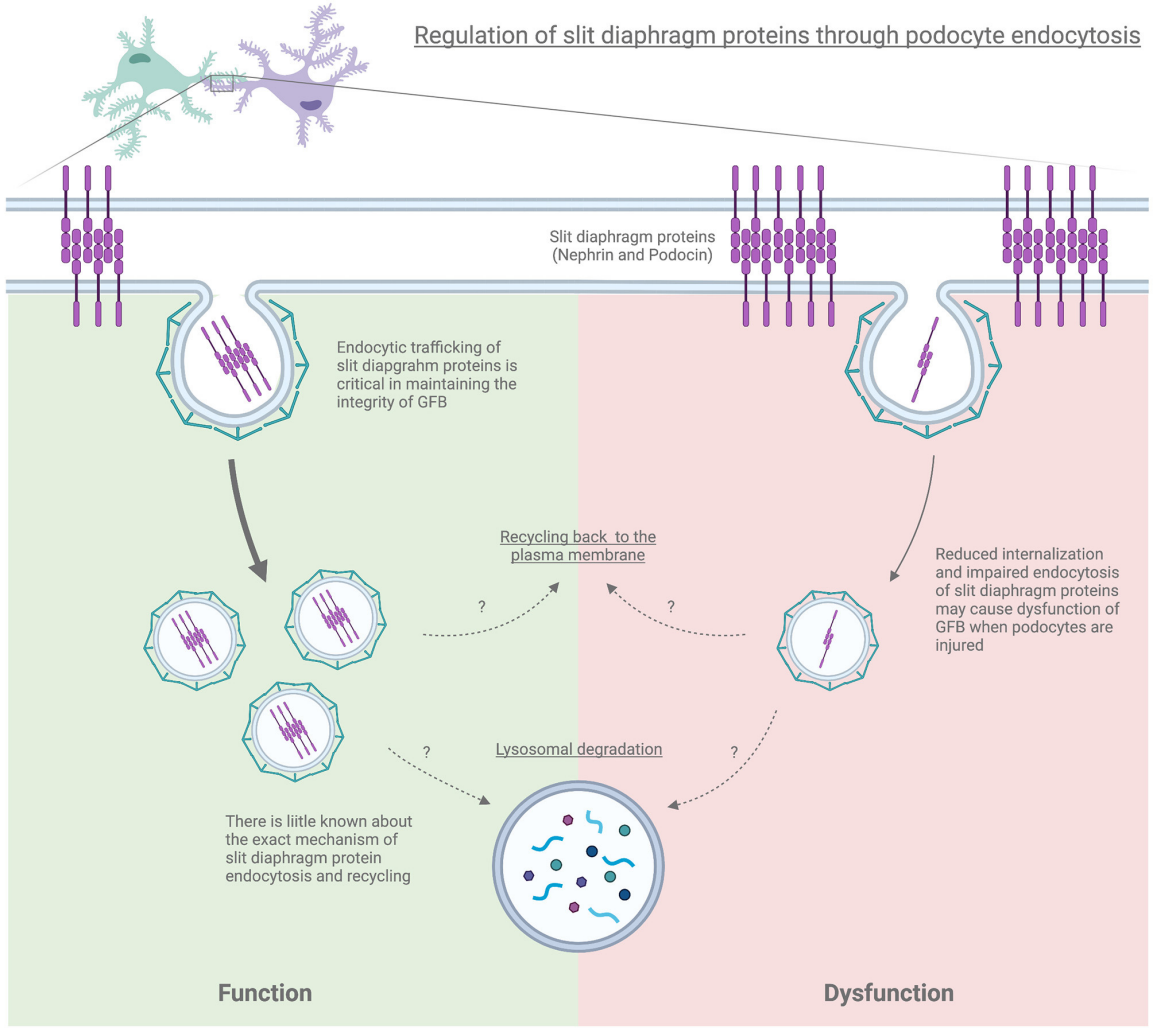
Regulation of slit diaphragm proteins through podocyte endocytosis.

Surface expression and turnover of podocin and nephrin appear to be intimately connected. A missense mutation in podocin cause retention of both podocin and nephrin in the cytoplasm (120, 121). Factors influencing nephrin and podocin turnover may destabilize the slit diaphragm resulting in subsequent disruption of the glomerular filtration barrier (Figure 2). However, precisely how disruption of endocytic trafficking cause defects in the slit diaphragm requires further investigation.

### Regulation of Filtered Proteins in Podocyte Endocytosis

Albuminuria is a hallmark of proteinuric chronic kidney disease and is usually caused by dysfunction of glomerular filtration barrier integrity. Interestingly, albumin endocytosis is enhanced in the podocytes from minimal change disease patients. Accumulation of blue-labeled albumin endocytic vesicles was observed in the podocytes in a rat model of puromycin aminonucleoside-induced nephrotic syndrome (122). Furthermore, overexpression of Myo1e may also enhance dynamin-mediated albumin endocytosis in mouse podocytes (90). Ang II enhances the migration of albumin-containing vesicles from the glomerular capillary to the apical aspect of the podocyte, which is eventually released into the urinary space. This increase in endocytosis and transcytosis of plasma albumin by podocytes, which is regulated by Ang II, may potentially play a critical role in the formation of proteinuria (123). The caveolae-dependent albumin endocytosis has been demonstrated in cultured immortalized human podocytes (39). Filtered albumin enters into the podocyte through Fc-receptors, and reaches the early endosome where albumin is sorted for lysosomal degradation or exocytosed outside of the podocytes (124).

Lipid endocytosis in podocytes has also been implicated to reduce cellular integrity and worsens proteinuria and kidney damage, such as in obesity-related glomerulopathy. Enhanced lipid endocytosis by podocytes from patients with obesity-related glomerulopathy was observed and was associated with decreased expression of phosphatase and tensin homolog (PTEN). Silencing of PTEN promoted low-density lipoprotein endocytosis in mouse podocytes, while PTEN overexpression inhibited the endocytosis of podocyte lipoproteins. PTEN directly dephosphorylates and activates the actin-depolymerizing factor cofilin-1, leading to depolymerization of F-actin, which is necessary for endocytosis. Inhibition of PTEN resulted in the phosphorylation and inactivation of cofilin-1, leading to F-actin formation that enhanced the endocytosis of lipoproteins in podocytes (125). Moreover, free fatty acid (FFA) associated with albumin has been shown to stimulate micropinocytosis through FFA receptors during Adriamycin-induced podocyte injury worsening albuminuria (126).

Mutations in *CLCN5*, encoding the chloride channel Cl^−^/H^+^ exchanger ClC-5, have been identified in FSGS patients with Dent disease using exome sequencing. CLCN5 has been shown also to be expressed in cultured human podocytes. Knockdown of *CLCN5* in human podocytes resulted in defective transferrin endocytosis, which suggests that CLCN5 plays a critical role in podocyte function by participating in endocytosis, although the role of targeted cargoes needs to be further investigated podocytes (127).

The apolipoprotein L1 (*APOL1*) gene (*APOL1*) variants such as its G1 and G2 alleles are associated with increased risk for nephropathy progression in African Americans including FSGS and human immunodeficiency virus-associated nephropathy (HIVAN). Exogenous recombinant APOL1 can be taken up by human podocytes *in vitro* via clathrin-dependent endocytosis, contributing to the accumulation of APOL1 in podocytes and may be partially involved in the *APOL1*-associated renal toxicity, particularly with the G1 and G2 disease variants (128). However, cytotoxicity and effective endocytosis through a clathrin-dependent pathway were not observed in HEK293T cells (129). This discrepancy may be related to the use of different cells or different experimental conditions. Thus, the role of APOL1 endocytosis in podocytes and *APOL1*-associated renal toxicity is worthy of further studies.

### Altered Cell Signaling Pathways Due to Defective Podocyte Endocytosis

Under hyperglycemic conditions, enhanced recruitment of membranous protein kinase C-α (PKC-α) facilitates epidermal growth factor receptor (EGFR) ubiquitination and endocytosis, resulting in podocyte extracellular signal-regulated kinase (ERK) induced cellular damage in diabetic kidney disease models. Inhibiting PKC-α or ubiquitin-activating enzyme activation attenuates EGFR internalization and mitigates the high-glucose-induced podocyte injury (130). Epsins, a family of membrane proteins acting as endocytic adaptors, also play a critical role in the CME and CIE. In mammals, there are three different epsins namely, epsin1 (encoded by *Epn1*), espin2(encoded by *Epn2*), and espin3(encoded by *Epn3*). Podocyte-specific loss of epsins resulted in increased albuminuria and foot process effacement. Primary podocytes isolated from these knockout mice exhibited abnormalities in cell adhesion and spreading, which can likely be attributed to reduced activation of cell division control protein 42 homolog (Cdc42) and serum response factor (SRF), resulting in diminished β1 integrin expression in the podocyte (27).

**Figure 3:**
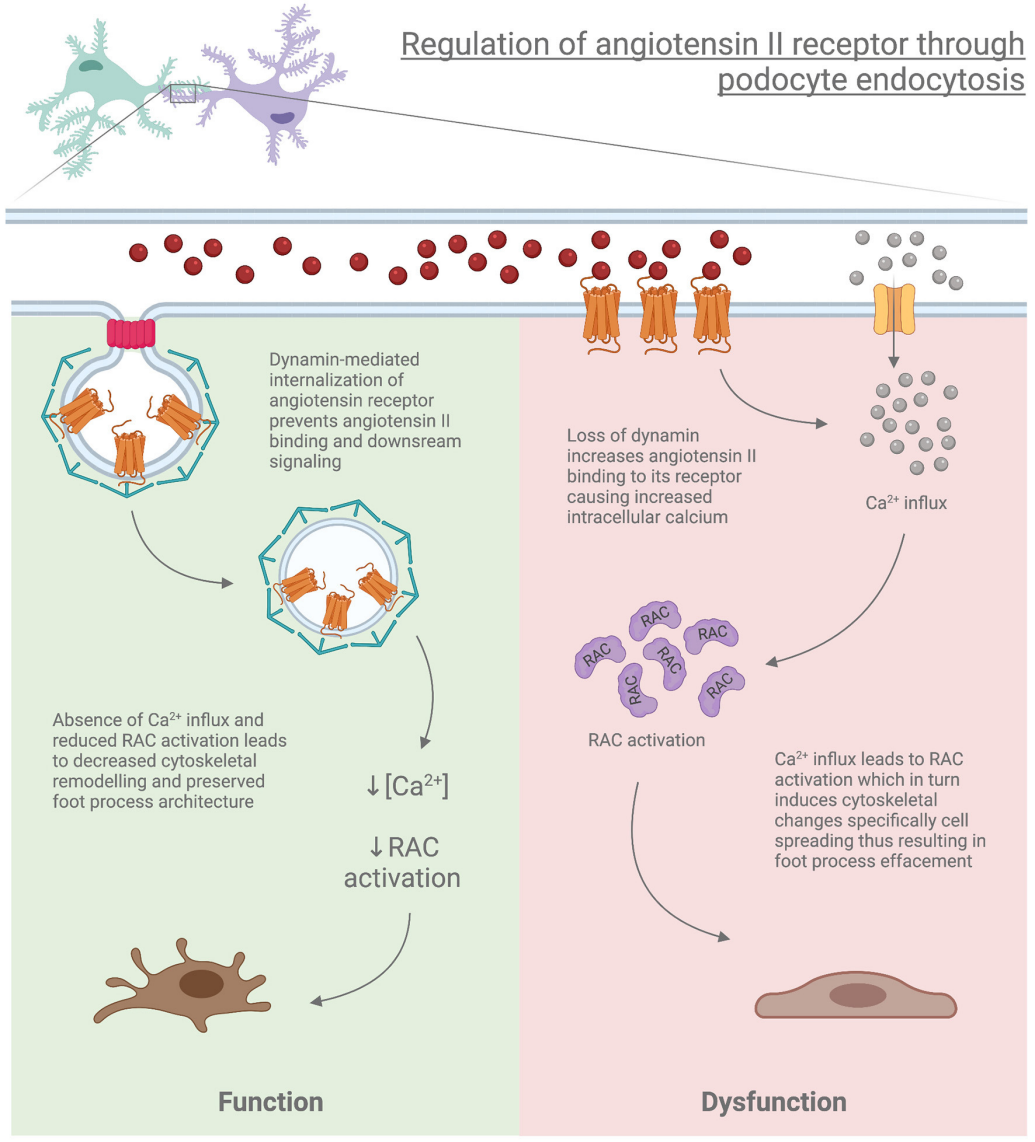
Regulation of angiotensin II receptor through podocyte endocytosis.

Another facet of the importance of dynamin and the endocytic process is through its role in the internalization of membrane-bound receptors such as Angiotensin II receptor type I (AT1R), which is internalized by CME. Podocyte-specific deletion of the AT1aR (Angiotensin II receptor type 1a which is mainly expressed in mice kidney) in *Dnm*-DKO mice demonstrated reduced albuminuria, improvement in both glomerulosclerosis and kidney function, and attenuation of membrane abnormalities. Furthermore, isolation of podocytes from *Dnm*-DKO mice revealed abnormal membrane dynamics, with increased Rac1 activation and lamellipodial extension, which was attenuated in *Dnm*-DKO podocytes lacking AT1aR (131). This suggests that loss of endocytic pathways may alter the internalization of receptors such as AT1aR, leading to the continued activation of this pathway contributing to worsened proteinuria and podocyte damage (Figure 3).

Clathrin-dependent and -independent pathways also contribute to β1 integrin endocytosis (132). Integrins are a family of transmembrane receptors that integrate cell adhesion to the extracellular matrix with a dynamic link of the cellular cytoskeleton and are important for a plethora of physiological and pathophysiological roles in podocytes. Podocyte-specific loss of integrin α3 or β1 in mice causes massive proteinuria and foot process effacement (133, 134). Integrins are subject to endocytosis and endosomal recycling back to the plasma membrane to maintain their turnover allowing for adaptive adhesive behavior responding to the microenvironment. PEA-15 (Phosphoprotein Enriched in Astrocytes of 15 kDa) is a member of the Death Effector Domain (DED) family, which can regulate α5β1 integrin endocytosis by interacting with β1 integrin cytoplasmic domains as a key endosomal adaptor, hence facilitating cell motility (135). Plasminogen activator inhibitor type-1 (PAI-1) / urokinase plasminogen activator (uPA) complex-mediated uPAR-dependent endocytosis of β1 integrin can also cause podocyte injury and progressive podocytes loss (136). The endocytic machinery for β1 integrin is potentially through clathrin- and dynamin-independent endocytic route (CLIC-GEEC) via swiprosin-1 (Swip1) and small GTPase Rab21 (137). These findings provide new insight into understanding the endocytic role in cell signaling and how it plays an important role in podocyte injury.

## Concluding Remarks

It has been established that podocyte proteins are uniquely organized to maintain the integrity of the GFB and its function. and genetic mouse models have validated its importance Table 1. However, the underlying mechanisms that participate in trafficking these proteins to the appropriate location in health and disease states need to be further studied in order to unravel complex pathophysiological relationships. Identifying these mechanisms will likely contribute to developing potential novel therapeutical targets to abate proteinuric CKD. Given the observations that neonatal Fc receptor (FcRn) expression in human podocytes is increased with proteinuric CKD, a nanoparticle-containing albumin and glucocorticoid molecules can be taken up by FcRn receptor-mediated endocytosis. This aims to decrease or avoid the systemic side effects caused by the clinical glucocorticoids administration (138). Moreover, with some of the most abundant podocyte-specific cargoes identified through mass spectrometry (139), future studies should enlighten critical pathways regulated by podocytes. In conclusion, these current cutting-edge findings on the relationship between endocytosis and podocyte biology will help us to further understand the precise mechanism of podocyte injury and provide impetus to find novel and useful therapeutic approaches to target abnormal endocytic machinery.

## Author Contributions

XT, PB, and SI all participated in writing the manuscript.

## Funding

This study was supported by the U.S. National Institute of Health grants DK083294 and DK093269 to SI.

## Conflict of Interest

The authors declare that the research was conducted in the absence of any commercial or financial relationships that could be construed as a potential conflict of interest. The handling Editor declared a shared affiliation with the authors at the time of review.

## Publisher's Note

All claims expressed in this article are solely those of the authors and do not necessarily represent those of their affiliated organizations, or those of the publisher, the editors and the reviewers. Any product that may be evaluated in this article, or claim that may be made by its manufacturer, is not guaranteed or endorsed by the publisher.

## References

[1] Ellinger I Pietschmann P. Endocytosis in health and disease-a thematic issue dedicated to Renate Fuchs. Wien Med Wochenschr. (2016) 166:193–5. 10.1007/s10354-016-0454-1 27165702

[2] Hermle T Schneider R Schapiro D Braun DA van der Ven AT Warejko JK. GAPVD1 and ANKFY1 mutations Implicate RAB5 regulation in nephrotic syndrome. J Am Soc Nephrol. (2018) 29:2123–38. 10.1681/ASN.2017121312 29959197 PMC6065084

[3] Li S Ghosh C Xing Y Sun Y. Phosphatidylinositol 4,5-bisphosphate in the control of membrane trafficking. Int J Biol Sci. (2020) 16:2761–74. 10.7150/ijbs.49665 33061794 PMC7545710

[4] Doherty GJ McMahon HT. Mechanisms of endocytosis. Annu Rev Biochem. (2009) 78:857–902. 10.1146/annurev.biochem.78.081307.110540 19317650

[5] Roth TF Porter KR. Yolk protein uptake in the oocyte of the mosquito aedes aegypti. L. J Cell Biol. (1964) 20:313–32. 10.1083/jcb.20.2.313 14126875 PMC2106398

[6] Yamada E. The fine structure of the gall bladder epithelium of the mouse. J Biophys Biochem Cytol. (1955) 1:445–58. 10.1083/jcb.1.5.445 13263332 PMC2229656

[7] Vaughan RB. The romantic rationalist: a study of elie metchnikoff. Med Hist. (1965) 9:201–15. 10.1017/S0025727300030702 14321564 PMC1033501

[8] Tauber AI. Metchnikoff and the phagocytosis theory. Nat Rev Mol Cell Biol. (2003) 4:897–901. 10.1038/nrm1244 14625539

[9] Schmid SL Sorkin A Zerial M. Endocytosis: past, present, and future. Cold Spring Harb Perspect Biol. (2014) 6:a022509. 10.1101/cshperspect.a022509 25359499 PMC4292149

[10] Mayor S Parton RG Donaldson JG. Clathrin-independent pathways of endocytosis. Cold Spring Harb Perspect Biol. (2014) 6:a016758. 10.1101/cshperspect.a016758 24890511 PMC4031960

[11] Hansen SH Sandvig K van Deurs B. The preendosomal compartment comprises distinct coated and noncoated endocytic vesicle populations. J Cell Biol. (1991) 113:731–41. 10.1083/jcb.113.4.731 1673969 PMC2288983

[12] Kirkham M Fujita A Chadda R Nixon SJ Kurzchalia TV Sharma DK. Ultrastructural identification of uncoated caveolin-independent early endocytic vehicles. J Cell Biol. (2005) 168:465–76. 10.1083/jcb.200407078 15668297 PMC2171740

[13] Tian X Ishibe S. Targeting the podocyte cytoskeleton: from pathogenesis to therapy in proteinuric kidney disease. Nephrol Dial Transplant. (2016) 31:1577–83. 10.1093/ndt/gfw021 26968197 PMC5039341

[14] Vart P Powe NR McCulloch CE Saran R Gillespie BW Saydah S. National trends in the prevalence of chronic kidney disease among racial/ethnic and socioeconomic status groups, 1988-2016. JAMA Netw Open. (2020) 3:e207932. 10.1001/jamanetworkopen.2020.7932 32672828 PMC7366187

[15] Dorval G Kuzmuk V Gribouval O Welsh GI Bierzynska A Schmitt A. TBC1D8B loss-of-function mutations lead to X-linked nephrotic syndrome via defective trafficking pathways. Am J Hum Genet. (2019) 104:348–55. 10.1016/j.ajhg.2018.12.016 30661770 PMC6369567

[16] Harris DP Vogel P Wims M Moberg K Humphries J Jhaver KG. Requirement for class II phosphoinositide 3-kinase C2alpha in maintenance of glomerular structure and function. Mol Cell Biol. (2011) 31:63–80. 10.1128/MCB.00468-10 20974805 PMC3019860

[17] Bechtel W Helmstadter M Balica J Hartleben B Kiefer B Hrnjic F. Vps34 deficiency reveals the importance of endocytosis for podocyte homeostasis. J Am Soc Nephrol. (2013) 24:727–43. 10.1681/ASN.2012070700 23492732 PMC3636793

[18] Chen J Chen MX Fogo AB Harris RC Chen JK. mVps34 deletion in podocytes causes glomerulosclerosis by disrupting intracellular vesicle trafficking. J Am Soc Nephrol. (2013) 24:198–207. 10.1681/ASN.2012010101 23291473 PMC3559479

[19] Venkatareddy M Verma R Kalinowski A Patel SR Shisheva A Garg P. Distinct requirements for vacuolar protein sorting 34 downstream effector phosphatidylinositol 3-phosphate 5-kinase in podocytes versus proximal tubular cells. J Am Soc Nephrol. (2016) 27:2702–19. 10.1681/ASN.2015050555 26825532 PMC5004647

[20] Soda K Balkin DM Ferguson SM Paradise S Milosevic I Giovedi S. Role of dynamin, synaptojanin, and endophilin in podocyte foot processes. J Clin Invest. (2012) 122:4401–11. 10.1172/JCI65289 23187129 PMC3533561

[21] Shih NY Li J Karpitskii V Nguyen A Dustin ML Kanagawa O. Congenital nephrotic syndrome in mice lacking CD2-associated protein. Science. (1999) 286:312–5. 10.1126/science.286.5438.312 10514378

[22] Tian X Inoue K Zhang Y Wang Y Sperati CJ Pedigo CE. Inhibiting calpain 1 and 2 in cyclin G associated kinase-knockout mice mitigates podocyte injury. JCI Insight. (2020) 5:e142740. 10.1172/jci.insight.142740 33208557 PMC7710277

[23] Teng B Schroder P Muller-Deile J Schenk H Staggs L Tossidou I. CIN85 deficiency prevents nephrin endocytosis and proteinuria in diabetes. Diabetes. (2016) 65:3667–79. 10.2337/db16-0081 27531950 PMC5314701

[24] Tossidou I Teng B Menne J Shushakova N Park JK Becker JU. Podocytic PKC-alpha is regulated in murine and human diabetes and mediates nephrin endocytosis. PLoS ONE. (2010) 5:e10185. 10.1371/journal.pone.0010185 20419132 PMC2855708

[25] Menne J Shushakova N Bartels J Kiyan Y Laudeley R Haller H. Dual inhibition of classical protein kinase C-alpha and protein kinase C-beta isoforms protects against experimental murine diabetic nephropathy. Diabetes. (2013) 62:1167–74. 10.2337/db12-0534 23434935 PMC3609593

[26] Nihalani D Solanki AK Arif E Srivastava P Rahman B Zuo X. Disruption of the exocyst induces podocyte loss and dysfunction. J Biol Chem. (2019) 294:10104–19. 10.1074/jbc.RA119.008362 31073028 PMC6664173

[27] Wang Y Pedigo CE Inoue K Tian X Cross E Ebenezer K. Murine epsins play an integral role in podocyte function. J Am Soc Nephrol. (2020) 31:2870–86. 10.1681/ASN.2020050691 33051360 PMC7790213

[28] Ramanan V Agrawal NJ Liu J Engles S Toy R Radhakrishnan R. Systems biology and physical biology of clathrin-mediated endocytosis. Integr Biol (Camb). (2011) 3:803–15. 10.1039/c1ib00036e 21792431 PMC3153420

[29] Kaksonen M Roux A. Mechanisms of clathrin-mediated endocytosis. Nat Rev Mol Cell Biol. (2018) 19:313–26. 10.1038/nrm.2017.132 29410531

[30] Gallop JL Jao CC Kent HM Butler PJ Evans PR Langen R. Mechanism of endophilin N-BAR domain-mediated membrane curvature. EMBO J. (2006) 25:2898–910. 10.1038/sj.emboj.7601174 16763559 PMC1500843

[31] Praefcke GJ Ford MG Schmid EM Olesen LE Gallop JL Peak-Chew SY. Evolving nature of the AP2 alpha-appendage hub during clathrin-coated vesicle endocytosis. EMBO J. (2004) 23:4371–83. 10.1038/sj.emboj.7600445 15496985 PMC526462

[32] Soda K Ishibe S. The function of endocytosis in podocytes. Curr Opin Nephrol Hypertens. (2013) 22:432–8. 10.1097/MNH.0b013e3283624820 23703394 PMC4143890

[33] McMahon HT Boucrot E. Molecular mechanism and physiological functions of clathrin-mediated endocytosis. Nat Rev Mol Cell Biol. (2011) 12:517–33. 10.1038/nrm3151 21779028

[34] Marks B Stowell MH Vallis Y Mills IG Gibson A Hopkins CR. GTPase activity of dynamin and resulting conformation change are essential for endocytosis. Nature. (2001) 410:231–5. 10.1038/35065645 11242086

[35] Charpentier JC King PD. Mechanisms and functions of endocytosis in T cells. Cell Commun Signal. (2021) 19:92. 10.1186/s12964-021-00766-3 34503523 PMC8427877

[36] Thomas B Matsushita K Abate KH Al-Aly Z Arnlov J Asayama K. Global cardiovascular and renal outcomes of reduced GFR. J Am Soc Nephrol. (2017) 28:2167–79. 10.1681/ASN.2016050562 28408440 PMC5491277

[37] Pelkmans L Helenius A. Endocytosis via caveolae. Traffic. (2002) 3:311–20. 10.1034/j.1600-0854.2002.30501.x 11967125

[38] Monier S Parton RG Vogel F Behlke J Henske A Kurzchalia TV. VIP21-caveolin, a membrane protein constituent of the caveolar coat, oligomerizes *in vivo* and *in vitro*. Mol Biol Cell. (1995) 6:911–27. 10.1091/mbc.6.7.911 7579702 PMC301248

[39] Dobrinskikh E Okamura K Kopp JB Doctor RB Blaine J. Human podocytes perform polarized, caveolae-dependent albumin endocytosis. Am J Physiol Renal Physiol. (2014) 306:F941–51. 10.1152/ajprenal.00532.2013 24573386 PMC4010685

[40] Kovtun O Tillu VA Ariotti N Parton RG Collins BM. Cavin family proteins and the assembly of caveolae. J Cell Sci. (2015) 128:1269–78. 10.1242/jcs.167866 25829513 PMC4379724

[41] Jing Z Wei-jie Y Yi-Feng ZG. Down-regulation of Wt1 activates Wnt/beta-catenin signaling through modulating endocytic route of LRP6 in podocyte dysfunction *in vitro*. Cell Signal. (2015) 27:1772–80. 10.1016/j.cellsig.2015.05.018 26049137

[42] Frick M Bright NA Riento K Bray A Merrified C Nichols BJ. Coassembly of flotillins induces formation of membrane microdomains, membrane curvature, and vesicle budding. Curr Biol. (2007) 17:1151–6. 10.1016/j.cub.2007.05.078 17600709

[43] Glebov OO Bright NA Nichols BJ. Flotillin-1 defines a clathrin-independent endocytic pathway in mammalian cells. Nat Cell Biol. (2006) 8:46–54. 10.1038/ncb1342 16341206

[44] Otto GP Nichols BJ. The roles of flotillin microdomains–endocytosis and beyond. J Cell Sci. (2011) 124(Pt 23):3933–40. 10.1242/jcs.092015 22194304

[45] Sabharanjak S Sharma P Parton RG Mayor S. GPI-anchored proteins are delivered to recycling endosomes via a distinct cdc42-regulated, clathrin-independent pinocytic pathway. Dev Cell. (2002) 2:411–23. 10.1016/S1534-5807(02)00145-4 11970892

[46] Chaudhary N Gomez GA Howes MT Lo HP McMahon KA Rae JA. Endocytic crosstalk: cavins, caveolins, and caveolae regulate clathrin-independent endocytosis. PLoS Biol. (2014) 12:e1001832. 10.1371/journal.pbio.1001832 24714042 PMC3979662

[47] Basquin C Trichet M Vihinen H Malarde V Lagache T Ripoll L. Membrane protrusion powers clathrin-independent endocytosis of interleukin-2 receptor. EMBO J. (2015) 34:2147–61. 10.15252/embj.201490788 26124312 PMC4557667

[48] Grassart A Dujeancourt A Lazarow PB Dautry-Varsat A Sauvonnet N. Clathrin-independent endocytosis used by the IL-2 receptor is regulated by Rac1, Pak1 and Pak2. EMBO Rep. (2008) 9:356–62. 10.1038/embor.2008.28 18344974 PMC2288760

[49] Inoue K Ishibe S. Podocyte endocytosis in the regulation of the glomerular filtration barrier. Am J Physiol Renal Physiol. (2015) 309:F398–405. 10.1152/ajprenal.00136.2015 26084928 PMC4556893

[50] Brown FD Rozelle AL Yin HL Balla T Donaldson JG. Phosphatidylinositol 4,5-bisphosphate and Arf6-regulated membrane traffic. J Cell Biol. (2001) 154:1007–17. 10.1083/jcb.200103107 11535619 PMC2196179

[51] Donaldson JG Radhakrishna H. Expression and properties of ADP-ribosylation factor (ARF6) in endocytic pathways. Methods Enzymol. (2001) 329:247–56. 10.1016/S0076-6879(01)29085-5 11210541

[52] Honda A Nogami M Yokozeki T Yamazaki M Nakamura H Watanabe H. Phosphatidylinositol 4-phosphate 5-kinase alpha is a downstream effector of the small G protein ARF6 in membrane ruffle formation. Cell. (1999) 99:521–32. 10.1016/S0092-8674(00)81540-8 10589680

[53] Rosales C Uribe-Querol E. Phagocytosis: a fundamental process in immunity. Biomed Res Int. (2017) 2017:9042851. 10.1155/2017/9042851 28691037 PMC5485277

[54] King JS Kay RR. The origins and evolution of macropinocytosis. Philos Trans R Soc Lond B Biol Sci. (2019) 374:20180158. 10.1098/rstb.2018.0158 30967007 PMC6304743

[55] Casamento A Boucrot E. Molecular mechanism of fast endophilin-mediated endocytosis. Biochem J. (2020) 477:2327–45. 10.1042/BCJ20190342 32589750 PMC7319585

[56] Doughman RL Firestone AJ Anderson RA. Phosphatidylinositol phosphate kinases put PI4,5P(2) in its place. J Membr Biol. (2003) 194:77–89. 10.1007/s00232-003-2027-7 14502432

[57] Bunney TD Katan M. Phosphoinositide signalling in cancer: beyond PI3K and PTEN. Nat Rev Cancer. (2010) 10:342–52. 10.1038/nrc2842 20414202

[58] Zoncu R Perera RM Sebastian R Nakatsu F Chen H Balla T. Loss of endocytic clathrin-coated pits upon acute depletion of phosphatidylinositol 4,5-bisphosphate. Proc Natl Acad Sci USA. (2007) 104:3793–8. 10.1073/pnas.0611733104 17360432 PMC1805489

[59] Cremona O Di Paolo G Wenk MR Luthi A Kim WT Takei K. Essential role of phosphoinositide metabolism in synaptic vesicle recycling. Cell. (1999) 99:179–88. 10.1016/S0092-8674(00)81649-9 10535736

[60] Perera RM Zoncu R Lucast L De Camilli P Toomre D. Two synaptojanin 1 isoforms are recruited to clathrin-coated pits at different stages. Proc Natl Acad Sci USA. (2006) 103:19332–7. 10.1073/pnas.0609795104 17158794 PMC1693868

[61] Mani M Lee SY Lucast L Cremona O Di Paolo G De Camilli P. The dual phosphatase activity of synaptojanin1 is required for both efficient synaptic vesicle endocytosis and reavailability at nerve terminals. Neuron. (2007) 56:1004–18. 10.1016/j.neuron.2007.10.032 18093523 PMC3653591

[62] Sauvonnet N Dujeancourt A Dautry-Varsat A. Cortactin and dynamin are required for the clathrin-independent endocytosis of gammac cytokine receptor. J Cell Biol. (2005) 168:155–63. 10.1083/jcb.200406174 15623579 PMC2171671

[63] Cao H Orth JD Chen J Weller SG Heuser JE McNiven MA. Cortactin is a component of clathrin-coated pits and participates in receptor-mediated endocytosis. Mol Cell Biol. (2003) 23:2162–70. 10.1128/MCB.23.6.2162-2170.2003 12612086 PMC149460

[64] Vidal-Quadras M Gelabert-Baldrich M Soriano-Castell D Llado A Rentero C Calvo M. Rac1 and calmodulin interactions modulate dynamics of ARF6-dependent endocytosis. Traffic. (2011) 12:1879–96. 10.1111/j.1600-0854.2011.01274.x 21883766

[65] Jarad G Miner JH. Update on the glomerular filtration barrier. Curr Opin Nephrol Hypertens. (2009) 18:226–32. 10.1097/MNH.0b013e3283296044 19374010 PMC2895306

[66] Li Q Gulati A Lemaire M Nottoli T Bale A Tufro A. Rho-GTPase activating protein myosin MYO9A identified as a novel candidate gene for monogenic focal segmental glomerulosclerosis. Kidney Int. (2021) 99:1102–17. 10.1016/j.kint.2020.12.022 33412162 PMC8076076

[67] Mele C Iatropoulos P Donadelli R Calabria A Maranta R Cassis P. MYO1E mutations and childhood familial focal segmental glomerulosclerosis. N Engl J Med. (2011) 365:295–306. 10.1056/NEJMoa1101273 21756023 PMC3701523

[68] Brown EJ Schlondorff JS Becker DJ Tsukaguchi H Tonna SJ Uscinski AL. Mutations in the formin gene INF2 cause focal segmental glomerulosclerosis. Nat Genet. (2010) 42:72–6. 10.1038/ng.505 20023659 PMC2980844

[69] Schneider R Deutsch K Hoeprich GJ Marquez J Hermle T Braun DA. DAAM2 variants cause nephrotic syndrome via actin dysregulation. Am J Hum Genet. (2020) 107:1113–28. 10.1016/j.ajhg.2020.11.008 33232676 PMC7820625

[70] Kaplan JM Kim SH North KN Rennke H Correia LA Tong HQ. Mutations in ACTN4, encoding alpha-actinin-4, cause familial focal segmental glomerulosclerosis. Nat Genet. (2000) 24:251–6. 10.1038/73456 10700177

[71] Kim JM Wu H Green G Winkler CA Kopp JB Miner JH. CD2-associated protein haploinsufficiency is linked to glomerular disease susceptibility. Science. (2003) 300:1298–300. 10.1126/science.1081068 12764198

[72] Hall G Lane BM Khan K Pediaditakis I Xiao J Wu G. The human FSGS-Causing ANLN R431C mutation induces dysregulated PI3K/AKT/mTOR/Rac1 signaling in podocytes. J Am Soc Nephrol. (2018) 29:2110–22. 10.1681/ASN.2017121338 30002222 PMC6065096

[73] Akamatsu M Vasan R Serwas D Ferrin MA Rangamani P Drubin DG. Principles of self-organization and load adaptation by the actin cytoskeleton during clathrin-mediated endocytosis. Elife. (2020) 9:e49840. 10.7554/eLife.49840 31951196 PMC7041948

[74] Merrifield CJ Qualmann B Kessels MM Almers W. Neural wiskott aldrich syndrome protein (N-WASP) and the Arp2/3 complex are recruited to sites of clathrin-mediated endocytosis in cultured fibroblasts. Eur J Cell Biol. (2004) 83:13–8. 10.1078/0171-9335-00356 15085951

[75] Collins A Warrington A Taylor KA Svitkina T. Structural organization of the actin cytoskeleton at sites of clathrin-mediated endocytosis. Curr Biol. (2011) 21:1167–75. 10.1016/j.cub.2011.05.048 21723126 PMC3143238

[76] Sever S Altintas MM Nankoe SR Moller CC Ko D Wei C. Proteolytic processing of dynamin by cytoplasmic cathepsin L is a mechanism for proteinuric kidney disease. J Clin Invest. (2007) 117:2095–104. 10.1172/JCI32022 17671649 PMC1934589

[77] Ferguson SM Raimondi A Paradise S Shen H Mesaki K Ferguson A. Coordinated actions of actin and BAR proteins upstream of dynamin at endocytic clathrin-coated pits. Dev Cell. (2009) 17:811–22. 10.1016/j.devcel.2009.11.005 20059951 PMC2861561

[78] Khalil R Koop K Kreutz R Spaink HP Hogendoorn PC Bruijn JA. Increased dynamin expression precedes proteinuria in glomerular disease. J Pathol. (2019) 247:177–85. 10.1002/path.5181 30350425 PMC6587474

[79] Altintas MM Reiser J. More expression, less function: cleaved dynamin in glomerular kidney disease. J Pathol. (2019) 247:413–5. 10.1002/path.5217 30549263

[80] Grassart A Cheng AT Hong SH Zhang F Zenzer N Feng Y. Actin and dynamin2 dynamics and interplay during clathrin-mediated endocytosis. J Cell Biol. (2014) 205:721–35. 10.1083/jcb.201403041 24891602 PMC4050722

[81] Krueger EW Orth JD Cao H McNiven MA. A dynamin-cortactin-Arp2/3 complex mediates actin reorganization in growth factor-stimulated cells. Mol Biol Cell. (2003) 14:1085–96. 10.1091/mbc.e02-08-0466 12631725 PMC151581

[82] Gu C Yaddanapudi S Weins A Osborn T Reiser J Pollak M. Direct dynamin-actin interactions regulate the actin cytoskeleton. EMBO J. (2010) 29:3593–606. 10.1038/emboj.2010.249 20935625 PMC2982766

[83] Schiffer M Teng B Gu C Shchedrina VA Kasaikina M Pham VA. Pharmacological targeting of actin-dependent dynamin oligomerization ameliorates chronic kidney disease in diverse animal models. Nat Med. (2015) 21:601–9. 10.1038/nm.3843 25962121 PMC4458177

[84] Gu C Lee HW Garborcauskas G Reiser J Gupta V Sever S. Dynamin autonomously regulates podocyte focal adhesion maturation. J Am Soc Nephrol. (2017) 28:446–51. 10.1681/ASN.2016010008 27432739 PMC5280009

[85] La TM Tachibana H Li SA Abe T Seiriki S Nagaoka H. Dynamin 1 is important for microtubule organization and stabilization in glomerular podocytes. FASEB J. (2020) 34:16449–63. 10.1096/fj.202001240RR 33070431

[86] Chandrasekar I Goeckeler ZM Turney SG Wang P Wysolmerski RB Adelstein RS. Nonmuscle myosin II is a critical regulator of clathrin-mediated endocytosis. Traffic. (2014) 15:418–32. 10.1111/tra.12152 24443954 PMC3975594

[87] Grahammer F Wigge C Schell C Kretz O Patrakka J Schneider S. A flexible, multilayered protein scaffold maintains the slit in between glomerular podocytes. JCI Insight. (2016) 1:e86177. 10.1172/jci.insight.86177 27430022 PMC4943462

[88] Cheng J Grassart A Drubin DG. Myosin 1E coordinates actin assembly and cargo trafficking during clathrin-mediated endocytosis. Mol Biol Cell. (2012) 23:2891–904. 10.1091/mbc.e11-04-0383 22675027 PMC3408416

[89] Navines-Ferrer A Martin M. Long-tailed unconventional class I myosins in health and disease. Int J Mol Sci. (2020) 21:2555. 10.3390/ijms21072555 32272642 PMC7177449

[90] Shen H Bao Y Feng C Fu H Mao J. Overexpression of Myo1e promotes albumin endocytosis by mouse glomerular podocytes mediated by Dynamin. PeerJ. (2020) 8:e8599. 10.7717/peerj.8599 32211226 PMC7083160

[91] Bywaters BC Rivera GM. Nck adaptors at a glance. J Cell Sci. (2021) 134:jcs258965. 10.1242/jcs.258965 34558601 PMC10999758

[92] Martin CE New LA Phippen NJ Keyvani Chahi A Mitro AE Takano T. Multivalent nephrin-Nck interactions define a threshold for clustering and tyrosine-dependent nephrin endocytosis. J Cell Sci. (2020) 133:jcs236877. 10.1242/jcs.236877 31974115

[93] Wunderlich L Farago A Downward J Buday L. Association of Nck with tyrosine-phosphorylated SLP-76 in activated T lymphocytes. Eur J Immunol. (1999) 29:1068–75. 10.1002/(SICI)1521-4141(199904)29:04&lt;1068::AID-IMMU1068&gt;3.0.CO;2-P 10229072

[94] Humphries AC Donnelly SK Way M. Cdc42 and the Rho GEF intersectin-1 collaborate with Nck to promote N-WASP-dependent actin polymerisation. J Cell Sci. (2014) 127(Pt 3):673–85. 10.1242/jcs.141366 24284073

[95] Gigante M Pontrelli P Montemurno E Roca L Aucella F Penza R. CD2AP mutations are associated with sporadic nephrotic syndrome and focal segmental glomerulosclerosis (FSGS). Nephrol Dial Transplant. (2009) 24:1858–64. 10.1093/ndt/gfn712 19131354

[96] Cummins TD Wu KZL Bozatzi P Dingwell KS Macartney TJ Wood NT. PAWS1 controls cytoskeletal dynamics and cell migration through association with the SH3 adaptor CD2AP. J Cell Sci. (2018) 131:jcs202390. 10.1101/106971 29175910 PMC5818054

[97] Lowik MM Groenen PJ Pronk I Lilien MR Goldschmeding R Dijkman HB. Focal segmental glomerulosclerosis in a patient homozygous for a CD2AP mutation. Kidney Int. (2007) 72:1198–203. 10.1038/sj.ki.5002469 17713465

[98] Park BC Yim YI Zhao X Olszewski MB Eisenberg E Greene LE. The clathrin-binding and J-domains of GAK support the uncoating and chaperoning of clathrin by Hsc70 in the brain. J Cell Sci. (2015) 128:3811–21. 10.1242/jcs.171058 26345367 PMC4631779

[99] Greener T Zhao X Nojima H Eisenberg E Greene LE. Role of cyclin G-associated kinase in uncoating clathrin-coated vesicles from non-neuronal cells. J Biol Chem. (2000) 275:1365–70. 10.1074/jbc.275.2.1365 10625686

[100] Boulant S Kural C Zeeh JC Ubelmann F Kirchhausen T. Actin dynamics counteract membrane tension during clathrin-mediated endocytosis. Nat Cell Biol. (2011) 13:1124–31. 10.1038/ncb2307 21841790 PMC3167020

[101] Swiatecka-Urban A. Membrane trafficking in podocyte health and disease. Pediatr Nephrol. (2013) 28:1723–37. 10.1007/s00467-012-2281-y 22932996 PMC3578983

[102] Bai L Zhuang J Zhang C Lu C Tian X Jiang H. Case report: the monogenic familial steroid-resistant nephrotic syndrome caused by a novel missense mutation of NPHS2 Gene A593C in a chinese family. Front Pediatr. (2021) 9:692727. 10.3389/fped.2021.692727 34631609 PMC8497038

[103] Fukuda H Hidaka T Takagi-Akiba M Ichimura K Oliva Trejo JA Sasaki Y. Podocin is translocated to cytoplasm in puromycin aminonucleoside nephrosis rats and in poor-prognosis patients with IgA nephropathy. Cell Tissue Res. (2015) 360:391–400. 10.1007/s00441-014-2100-9 25676004 PMC4544490

[104] Sasaki Y Hidaka T Ueno T Akiba-Takagi M Oliva Trejo JA Seki T. Sorting Nexin 9 facilitates podocin endocytosis in the injured podocyte. Sci Rep. (2017) 7:43921. 10.1038/srep43921 28266622 PMC5339724

[105] Patrakka J Kestila M Wartiovaara J Ruotsalainen V Tissari P Lenkkeri U. Congenital nephrotic syndrome (NPHS1): features resulting from different mutations in Finnish patients. Kidney Int. (2000) 58:972–80. 10.1046/j.1523-1755.2000.00254.x 10972661

[106] Ruotsalainen V Ljungberg P Wartiovaara J Lenkkeri U Kestila M Jalanko H. Nephrin is specifically located at the slit diaphragm of glomerular podocytes. Proc Natl Acad Sci U S A. (1999) 96:7962–7. 10.1073/pnas.96.14.7962 10393930 PMC22170

[107] Tossidou I Teng B Drobot L Meyer-Schwesinger C Worthmann K Haller H. CIN85/RukL is a novel binding partner of nephrin and podocin and mediates slit diaphragm turnover in podocytes. J Biol Chem. (2010) 285:25285–95. 10.1074/jbc.M109.087239 20457601 PMC2919091

[108] Dumont V Tolvanen TA Kuusela S Wang H Nyman TA Lindfors S. PACSIN2 accelerates nephrin trafficking and is up-regulated in diabetic kidney disease. FASEB J. (2017) 31:3978–90. 10.1096/fj.201601265R 28550045 PMC5572687

[109] Teng B Duong M Tossidou I Yu X Schiffer M. Role of protein kinase C in podocytes and development of glomerular damage in diabetic nephropathy. Front Endocrinol (Lausanne). (2014) 5:179. 10.3389/fendo.2014.00179 25414693 PMC4220730

[110] Martin CE Petersen KA Aoudjit L Tilak M Eremina V Hardy WR. ShcA adaptor protein promotes nephrin endocytosis and is upregulated in proteinuric nephropathies. J Am Soc Nephrol. (2018) 29:92–103. 10.1681/ASN.2017030285 29018139 PMC5748910

[111] Liu Y Su H Ma C Ji D Zheng X Wang P. IQGAP1 mediates podocyte injury in diabetic kidney disease by regulating nephrin endocytosis. Cell Signal. (2019) 59:13–23. 10.1016/j.cellsig.2019.03.009 30857827

[112] Espiritu EB Jiang H Moreau-Marquis S Sullivan M Yan K Beer Stolz D. The human nephrin Y (1139) RSL motif is essential for podocyte foot process organization and slit diaphragm formation during glomerular development. J Biol Chem. (2019) 294:10773–88. 10.1074/jbc.RA119.008235 31152064 PMC6635437

[113] Jones N Blasutig IM Eremina V Ruston JM Bladt F Li H. Nck adaptor proteins link nephrin to the actin cytoskeleton of kidney podocytes. Nature. (2006) 440:818–23. 10.1038/nature04662 16525419

[114] Feng D. Phosphorylation of key podocyte proteins and the association with proteinuric kidney disease. Am J Physiol Renal Physiol. (2020) 319:F284–F91. 10.1152/ajprenal.00002.2020 32686524 PMC7528399

[115] Qin XS Tsukaguchi H Shono A Yamamoto A Kurihara H Doi T. Phosphorylation of nephrin triggers its internalization by raft-mediated endocytosis. J Am Soc Nephrol. (2009) 20:2534–45. 10.1681/ASN.2009010011 19850954 PMC2794235

[116] Heiden S Siwek R Lotz ML Borkowsky S Schroter R Nedvetsky P. Apical-basal polarity regulators are essential for slit diaphragm assembly and endocytosis in Drosophila nephrocytes. Cell Mol Life Sci. (2021) 78:3657–72. 10.1007/s00018-021-03769-y 33651172 PMC8038974

[117] Itoh M Nakadate K Matsusaka T Hunziker W Sugimoto H. Effects of the differential expression of ZO-1 and ZO-2 on podocyte structure and function. Genes Cells. (2018) 23:546–56. 10.1111/gtc.12598 29845705

[118] Lausecker F Tian X Inoue K Wang Z Pedigo CE Hassan H. Vinculin is required to maintain glomerular barrier integrity. Kidney Int. (2018) 93:643–55. 10.1016/j.kint.2017.09.021 29241625 PMC5846847

[119] Mysh M Poulton JS. The basolateral polarity module promotes slit diaphragm formation in drosophila nephrocytes, a model of vertebrate podocytes. J Am Soc Nephrol. (2021) 32:1409–24. 10.1681/ASN.2020071050 33795424 PMC8259641

[120] Nishibori Y Liu L Hosoyamada M Endou H Kudo A Takenaka H. Disease-causing missense mutations in NPHS2 gene alter normal nephrin trafficking to the plasma membrane. Kidney Int. (2004) 66:1755–65. 10.1111/j.1523-1755.2004.00898.x 15496146

[121] Philippe A Weber S Esquivel EL Houbron C Hamard G Ratelade J. A missense mutation in podocin leads to early and severe renal disease in mice. Kidney Int. (2008) 73:1038–47. 10.1038/ki.2008.27 18288100

[122] Tojo A Hatakeyama S Kinugasa S Fukuda S Sakai T. Enhanced podocyte vesicle transport in the nephrotic rat. Med Mol Morphol. (2017) 50:86–93. 10.1007/s00795-016-0151-6 28314927

[123] Schiessl IM Hammer A Kattler V Gess B Theilig F Witzgall R. Intravital imaging reveals angiotensin ii-induced transcytosis of albumin by podocytes. J Am Soc Nephrol. (2016) 27:731–44. 10.1681/ASN.2014111125 26116357 PMC4769192

[124] Moriyama T Hasegawa F Miyabe Y Akiyama K Karasawa K Uchida K. Intracellular trafficking pathway of albumin in glomerular epithelial cells. Biochem Biophys Res Commun. (2021) 574:97–103. 10.1016/j.bbrc.2021.08.043 34450430

[125] Shi Y Wang C Zhou X Li Y Ma Y Zhang R. Downregulation of PTEN promotes podocyte endocytosis of lipids aggravating obesity-related glomerulopathy. Am J Physiol Renal Physiol. (2020) 318:F589–F99. 10.1152/ajprenal.00392.2019 31813249

[126] Chung JJ Huber TB Godel M Jarad G Hartleben B Kwoh C. Albumin-associated free fatty acids induce macropinocytosis in podocytes. J Clin Invest. (2015) 125:2307–16. 10.1172/JCI79641 25915582 PMC4518691

[127] Solanki AK Arif E Morinelli T Wilson RC Hardiman G Deng P. A novel CLCN5 mutation associated with focal segmental glomerulosclerosis and podocyte injury. Kidney Int Rep. (2018) 3:1443–53. 10.1016/j.ekir.2018.06.003 30426109 PMC6224352

[128] Ma L Shelness GS Snipes JA Murea M Antinozzi PA Cheng D. Localization of APOL1 protein and mRNA in the human kidney: nondiseased tissue, primary cells, and immortalized cell lines. J Am Soc Nephrol. (2015) 26:339–48. 10.1681/ASN.2013091017 25012173 PMC4310650

[129] Khatua AK Cheatham AM Kruzel ED Singhal PC Skorecki K Popik W. Exon 4-encoded sequence is a major determinant of cytotoxicity of apolipoprotein L1. Am J Physiol Cell Physiol. (2015) 309:C22–37. 10.1152/ajpcell.00384.2014 25924622 PMC4490327

[130] Lei CT Wei YH Tang H Wen Q Ye C Zhang C. PKC-alpha triggers EGFR ubiquitination, endocytosis and ERK activation in podocytes stimulated with high glucose. Cell Physiol Biochem. (2017) 42:281–94. 10.1159/000477329 28535513

[131] Inoue K Tian X Velazquez H Soda K Wang Z Pedigo CE. Inhibition of endocytosis of clathrin-mediated Angiotensin II receptor type 1 in podocytes augments glomerular injury. J Am Soc Nephrol. (2019) 30:2307–20. 10.1681/ASN.2019010053 31511362 PMC6900791

[132] Soriano-Castell D Chavero A Rentero C Bosch M Vidal-Quadras M Pol A. ROCK1 is a novel Rac1 effector to regulate tubular endocytic membrane formation during clathrin-independent endocytosis. Sci Rep. (2017) 7:6866. 10.1038/s41598-017-07130-x 28761175 PMC5537229

[133] Kreidberg JA Donovan MJ Goldstein SL Rennke H Shepherd K Jones RC. Alpha 3 beta 1 integrin has a crucial role in kidney and lung organogenesis. Development. (1996) 122:3537–47. 10.1242/dev.122.11.3537 8951069

[134] Pozzi A Jarad G Moeckel GW Coffa S Zhang X Gewin L. Beta1 integrin expression by podocytes is required to maintain glomerular structural integrity. Dev Biol. (2008) 316:288–301. 10.1016/j.ydbio.2008.01.022 18328474 PMC2396524

[135] Caliva MJ Yang WS Young-Robbins S Zhou M Yoon H Matter ML. Proteomics analysis identifies PEA-15 as an endosomal phosphoprotein that regulates alpha5beta1 integrin endocytosis. Sci Rep. (2021) 11:19830. 10.1038/s41598-021-99348-z 34615962 PMC8494857

[136] Kobayashi N Ueno T Ohashi K Yamashita H Takahashi Y Sakamoto K. Podocyte injury-driven intracapillary plasminogen activator inhibitor type 1 accelerates podocyte loss via uPAR-mediated beta1-integrin endocytosis. Am J Physiol Renal Physiol. (2015) 308:F614–26. 10.1152/ajprenal.00616.2014 25587125 PMC4360033

[137] Moreno-Layseca P Jantti NZ Godbole R Sommer C Jacquemet G Al-Akhrass H. Cargo-specific recruitment in clathrin- and dynamin-independent endocytosis. Nat Cell Biol. (2021) 23:1073–84. 10.1101/2020.10.05.323295 34616024 PMC7617174

[138] Wu L Chen M Mao H Wang N Zhang B Zhao X. Albumin-based nanoparticles as methylprednisolone carriers for targeted delivery towards the neonatal Fc receptor in glomerular podocytes. Int J Mol Med. (2017) 39:851–60. 10.3892/ijmm.2017.2902 28259932 PMC5360426

[139] Groener M WY Cross E Tian X Ebenezer K Baik E Pedigo CE. Identification of podocyte cargo proteins by proteomic analysis of clathrin-coated vesicles. Kidney360. (2020) 1:480–90. 10.34067/KID.0000212020 PMC8809311 35368594

